# The Relationship of Physiology Pharmacology, Pathology, and Practical Medicine*Read at the International Medical Congress at Moscow.

**Published:** 1898-01

**Authors:** T. Lauder Brunton


### THE RELATIONSHIP OF PHYSIOLOGY PHARMACOLOGY, PATHOLOGY, AND PRACTICAL MEDICINE.*

BY T. LAUDER BRUNTON. M. D., LL.D.

The desire for knowledge which is common to the lower animals and man, savage or civilized, and which has induced members of this Congress to come from the ends of the earth in order to gain information, must have led primitive man from the earliest times to study the great problems of physiology, the nature of life, of growth, of reproduction, and of death, as well as to notice the connection of the latter with mechanical injuries, such as the wounds inflicted by clubs and spears or by the teeth or claws of wild beasts. Next to the problems of physiology come those of pharmacology by which I mean the poisonous or remedial action of various substances—mineral, vegetable, or animal. A knowledge of this subject is found even amongst the lowest savages and is of the greatest use to them, for it enables them on the one hand to avoid eating things which may cause discomfort, pain, or death, and on the other, to obtain food by poisoning the waters and thus catching fish, or by poisoning their arrows to kill game which would otherwise escape.

Closely associated with the knowledge of the poisonous is that of the curative powers of herbs, and it is possessed by animals as well as man, for cows avoid noxious plants, and dogs will every now and again eat grass apparently as medicine. Primitive people use various substances as remedies in disease with more or less success, and one of the most extraordinary points in their practice is that they seem to some extent to have forestalled the newest researches on venins, antivenins, and organotherapy, for in Africa the bushmen are accustomed to drink the poison of venomous snakes as a prophylactic against their bite, and the Hausas prevent hydrophobia by kill-

*Read at the International Medical Congress at Moscow.

ing the mad dog and making the man it has bitten eat its liver. The occurrence of death from wounds or poison is intelligible even to a savage, but when illness and death occur independently of these, men naturally attribute them to invisible powers. Thus the Dyaks of Borneo ascribe sickness to wounds from invisible spears wielded by invisible spirits, and during an epidemic of disease in the middle ages the cry often arose that the wells had been poisoned. These crude ideas often contain germs of truth, and when we look at Professor Metchnikoff's drawings of a Daphnia attacked by a Monospora we seem to recognize the invisible darts of the Dyaks; while during an epidemic of typhoid fever we have often to acknowledge that our wells have been poisoned by bacilli.

### EVOLUTION OF PRIMITIVE SCIENCE.

It is impossible to trace the steps by which the crude ideas of savage peoples regarding physiology, pharmacology, and pathology have grown into definite sciences, or even to indicate the most important landmarks, though we naturally think of the names of Alkmaon, Galen, and Harvey in physiology; of Nicander, Magendie, and Bernard in pharmacology; and of Morgagni, Virchow, and Pasteur in pathology. During this century these three sciences have developed with almost incredible rapidity; a complete knowledge of them is enough to tax severely the most retentive memory; and it is almost impossible for any individual to keep up with the advance of all three of them. But just as the whole subject of astronomy became suddenly simplified by a change of standpoint at the very time when cycles and epicycles became most bewildering, so at the very time when these three sciences are becoming most complex and diverse they appear to be tending to unification and simplification. Pathology, for example, is now becomingto a great extent a branch of pharmacology, for while a few years ago its chief object was to discover, examine and classify the microbes which give rise to disease it is now striving rather to discover the nature and actions of the ferments and poisons which they form, and by which they are able to cause disease and death in the animals they attack. Pharmacological investigation, instead of being confined to the alkaloids and other poisons formed by higher plants, has now extended to those formed by microscopic plants or microbes, and thus comes to include a greater part of pathology. In the same way, though pharmacology is a branch of physiology, inasmuch as it deals with the phenomena of life as modified by drugs, yet physiology may to a certain extent be regarded as a branch of pharmacology, because some of the latest researches regarding the processes of life have been made by pharmacological methods, using the products of animal life instead of vegetable poisons. Amongst the pioneers in this line I may mention my two masters, Kuhne and Ludwig, the former of whom by his chemical investigations has enabled us to differentiate the various products of albuminous decomposition, whilst the latter, with his pupils Schmidt-Muhlheim and Woodbridge, discovered the poisonous action of albumoses and peptones, and of the juices of various tissues when injected directly into the blood.

INTERESTING PROBLEMS IN DIGESTION.

Before the proteid constituents of our food can be absorbed they must be split up during digestion into albumoses and peptones; yet these researches show that the very substances which are necessary to repair waste and are indispensable for the continuace of life prove fatal when introduced into the body in a wrong way or in too great quantity. But the products of the digestion of albumen do not normally enter the circulation as albumoses and peptones. During absorption they undergo changes of a synthetic nature in the walls of the intestine, and probably to a certain extent also in the liver, so that they again form harmless substances, and their poisonous properties are destroyed before they enter the general blood stream. But how is it that the ferments which decompose albuminous food and form poisons from it in the intestines do not pass into the blood and kill the animal by digesting the tissues and forming poisons from them? Of course pepsin can not do so, as it only acts in an acid medium, but there is no such hindrance to the action of trypsin, and yet it does not destroy the tissues composing the body itself. In all probability the reason why digestive ferments do not digest the tissues is not that they are destroyed in the digestive canal nor that they are not absorbed, but that they are altered from active enzymes into inert zymogens which can be stored up without risk, and can again liberate active enzymes when these are required to digest a subsequent meal. In this respect they may be compared to the knives used by wandering people to cut up their meat, and which are not thrown away after each meal, but are simply put into sheaths which cover their edges and deprive them for a time of their cutting power.

LIFE A PROCESS OF FERMENTATION·

But it is not in the intestine only that enzymes are found; they are also poured into the blood by the pancreas and probably by the thyroid and other glands. As our acquaintance with the processes of cell life increases it seems more and more likely that the tissue change on which functional activity depends is effected by enzymes, and the truer do the speculations of Van Helmont appear—that life is a process of fermentation. There can be little doubt that if enzymes in a free state were to circulate through the body they would do much harm, and indeed we may regard this as well-nigh proved in regard to the enzyme of tetanus. But their action is limited either by their conversion in zymogens or their localization to the cells or tissues where their action is required. This is more readily seen in plants than in animals, and one of the best examples of it is that in germinating wheat. In the ordinary state of the grain the diastatic ferment is kept apart from the starch by a small layer of cellulose, through which the diastase can not pass, but during germination another ferment appears which has the power of dissolving cellulose, and by breaking down this dividing membrane it allows the diastatic ferment to act upon the starch, and renders it available for the needs of the growing plant.

### EXTRACTION OF ENZYMES.

Enzymes appear to differ among themselves nearly as much as albumen, albumoses, and peptones. Some are easily separated from the cells in which they exist, whilst others are so closely united to the protoplasm that their separate existence apart from it has been denied. The yeast plant, for example, yields an invert enzyme which can be extracted with comparative ease, but the enzyme which splits up sugar into alcohol and carbonic acid is so firmly attached to the protoplasm of the cell that it is only within the last few months that it has been isolated by Buchner by the application of enormous presture. It is probable that the enzymes contained in the cells of animal tissues differ in like manner, and that by the use of similiar methods we may obtain a number of enzymes with which we are at present unacquainted. But it is not merely the products formed in the digestive canal or in the organs of animals during life, or even the alkaloids that are formed by the higher plants, that act as poisons. The processes of life are much the same in the lowest microbes as in animals or in the higher plants, and these microbes, by forming ferments and poisons, give rise to disturbance of function or death in animals. When grown in suitable media outside the body they produce enzymes and poisons, albumoses and alkaloids, and many of them continue to do so after their introduction into the body.

### POISON AND ANTIDOTE.

One of the most curious points, both in the chemistry of the higher plants and of microbes, is that they tend to form at the same time a poison and its antidote. In Calabar bean, for example we find there are two poisons—physostigmine and calabarine, the former tending to paralyze the spinal cord and the latter to stimulate it, so that each poison to a certain extent antagonizes the other. The same condition is found even more markedly in jaborandi, of which the two alkaloids, pilocarpine and jaborine, antagonize one another's action so that, although pilocarpine generally greatly predominates, it might be possible to get a specimen of the leaf *having no action at all*, although it contained a quantity of alkaloids. When injected into animals the toxins formed by microbes and the venins of serpents cause the production of antitoxins and antivenins, which neutralize their action apparently by chemical combination in somewhat the same way as an acid and alkali, each poisonous by itself, combine to form a comparatively inert salt: But the two components here, like an organic acid and a mineral base, are unequally affected by destructive agencies, and the antivenin may be destroyed, so that the venin again regains its activity. The conversion of zymogens into enzymes may be compared to the freeing of venins from their compounds, while the conversion of active venins into inert bodies by combination with antivenins suggests that a similar process may occur in the case of active enzymes, by which they may be converted into inactive zymogens.

Perhaps the hypothesis I mentioned eight years ago to my pupil and friend, Mr. Hankin, that the germicidal power of organisms is proportional to their power to produce enzymes, may not be altogether unfounded, and possibly we may discover also that immunity, natural or acquired, is nothing more than an extension to the cells of the tissues generally of a power which is constantly exercised during digestion by those of the intestine and liver. The problem is one which pertains to all three sciences and has a most important bearing on practical medicine.

### THE SOURCE OF MEDICAL PROGRESS.

Practical medicine, except when empirical, depends for its advance on physiology, pharmacology, and pathology. A knowledge of the physiology of digestion has led to the satisfactory treatment of dyspepsia by the administration of digestive enzymes, and pharmacological research has enabled us to treat diseases of the circulation with a success previously undreamed of, by teaching us not only how to use aright old remedies, such as digitalis, but also how to apply new ones, such as strophanthus and amyl nitrite, and even to manufacture others, such as nitro-erythol, which possess the special actions we desire but which are lacking in the drugs we already have. Indeed, new remedies which shall alter tissue change, lower temperature, relieve pain, and procure sleep, are now being made in such numbers that it is hard to keep count of them. But among all the new gains of practical medicine none are so remarkable as those which we owe to pathology. Time would fail me to speak of the prevention and cure of zymotic diseases, but no less astonishing is the discovery that myxedema depends on inactivity or absence of the thyroid gland, and can be cured by the administration of its extract, which seems to act as an enzyme on living tissues, so that the heavy, shapeless, features of the patient resume their natural expression, and the sluggish mental processes quicken.

An exhaustive study of enzymes and their products appears to be the most promising way of advancing our knowledge both of the nature and treatment of disease. Probably more is to be hoped for from an investigation into the nature and properties of those enzymes which are intimately associated with the protoplasm of the cells in the various tissues and organs than even of those which are poured into the blood by glands having an internal secretion, such as the thyroid. For all organs, even those which, like muscles and nerves, are not glandular, have an action on the blood comparable to that of the yeast plant, which modifies the fluid in which it lives by the substance which it removes from or adds to it. It is to a knowledge of the processes which occur in the protoplasm of the cells in the intestinal wall and liver, and of the enzymes by which these processes are in all probability carried out, that we must look for an explanation of the conversion of the poisonous albumoses formed during digestion into innocuous albumens, and of dangerous enzymes into harmless zymogens. Moreover it seems to me that it is by researches into the nature and action of the enzymes, not only of microbes, but in the various tissues of the body in higher animals, that we shall learn how the microbes, like the enzymes of the intestinal canal, produce poisonous albumoses and how the tissues, like the cells of the intestinal walls or liver, convert them into harmless or even protective substances. In this way we may hope to obtain an explanation of toxins and antitoxins, of pathogenesis and immunity, as well as the nature of diseases unconnected with the presence of microbes, such as diabetes. Twenty-three years ago I attempted to obtain a glycolytic enzyme from muscle, in order to enable diabetic patients to utilize the sugar in their blood. My attempt was unsuccessful, but we may still hope that by other methods we may obtain from animal organs various enzymes, the administration of which may prove as useful in other diseases as the thyroid glands in myxedema.

Practical medicine depends on physiology, pharmacology, and pathology, but all three are tending to become more and more subdivisions of the wider and all-embracing science of chemistry. It is to a chemist, Pasteur, that we owe the wonderful development of pathology within the last quarter of a century, and we may fairly regard his fellow-countryman, Lavoisier, as the founder of this science. Men from all countries, and especially from Germany, have aided its development; but it seems fitting that at this Congress, in acknowledging our obligations to this science, we should not omit to mention that at its head now stands a Russian, Mendeleef, whose marvelous prescience enabled him to predict the existence of elements which were then unknown, and even to describe their properties more correctly than those who first verified his predictions by obtaining the substances themselves. When we consider that little more than a hundred years have elapsed since the time of Lavoisier, and contemplate the vast benefits which medicine and its allied sciences have derived from chemistry during this time, our hopes can not be otherwise than great for centuries to come.—*Indian Lancet.*

